# The Effect and Mechanism of Transcranial Direct Current Stimulation on Episodic Memory in Patients With Mild Cognitive Impairment

**DOI:** 10.3389/fnins.2022.811403

**Published:** 2022-02-17

**Authors:** Jun Gu, Da Li, Zhaohui Li, Yuan Guo, Fuqiang Qian, Ying Wang, Li Tang

**Affiliations:** ^1^Department of Mental Rehabilitation, Wuxi Mental Health Center, Wuxi, China; ^2^Department of Neurorehabilitation, Wuxi Tongren Rehabilitation Hospital, Wuxi, China; ^3^Psychometric Laboratory, Wuxi Mental Health Center, Wuxi, China; ^4^Medical Administration Department, Wuxi Mental Health Center, Wuxi, China; ^5^Department of Psychiatry, Wuxi Mental Health Center, Wuxi, China

**Keywords:** event related potential (ERP), mild cognitive impairment (MCI), Montreal Cognitive Assessment Scale (MoCA), transcranial direct current stimulation (tDCS), Wechsler Memory Scale (WMS)

## Abstract

**Objective:**

This study aimed to investigate the efficacy of transcranial direct current stimulation (tDCS) on episodic memory in patients with mild cognitive impairment (MCI) and analyze the neural mechanism of tDCS therapy from the perspective of neuroelectrophysiological parameters.

**Methods:**

Forty MCI patients were recruited and randomly divided into a sham group (*n* = 20) and a tDCS group (*n* = 20). Patients in the tDCS group were treated with a tDCS instrument for 20 min, once a day, for 5 days. Patients in the sham group were treated with sham stimulus. Montreal Cognitive Assessment Scale (MoCA), Wechsler Memory Scale (WMS), and event-related potential (ERP) (amplitude and latency of P300 wave) were comparatively assessed between the two groups at pre-treatment, 5 days and 4 weeks post-treatment points.

**Results:**

The two groups showed no significant difference in any of the assessed parameters at pre-treatment (*P* > 0.05). At 5 days post-treatment, memory quotient (MQ) score in the tDCS group significantly increased (*P* < 0.05), scores of picture memory, visual regeneration, logical memory, memory span, visual regeneration-delay, and logical memory-delay were significantly increased compared to pre-treatment (*P* < 0.01). The P300 amplitude significantly increased, and its latency significantly shortened (*P* < 0.01). Four weeks post-treatment, the scores of MQ and visual regeneration-delay in the tDCS group increased, compared to pre-treatment (*P* < 0.05); picture memory, visual regeneration, logical memory, memory span, and logical memory-delay improved (*P* < 0.01); the P300 amplitude increased, and its latency shortened (*P* < 0.01). At 5 days and 4 weeks post-treatment points, the tDCS group, compared with the sham group (*P* < 0.01), exhibited greater scores of MQ, picture memory, visual regeneration, logical memory, memory span, visual regeneration-delay, and logical memory-delay, increased P300 amplitude, and shortened P300 latency. Similarly, the tDCS group showed higher MQ scores at 5 days post-treatment (*P* < 0.05) and 4 weeks post-treatment (*P* < 0.01). Before treatment and after 5 days of treatment, P300 amplitude and latency difference were positively correlated with MQ difference (*P* < 0.05).

**Conclusion:**

tDCS improved episodic memory in MCI patients, and the effect lasted for 4 weeks. Changes in ERP (P300) suggested that tDCS could promote changes in brain function.

## Introduction

Mild cognitive impairment (MCI) is an early stage of memory loss or deficits in other cognitive functions such as visual/spatial perception or language in individuals who are still able to independently perform their daily living activities. MCI is manifested as memory impairment and may be accompanied by cognitive impairment in attention, visual space, executive function, and language, but it does not affect daily life as a whole (Petersen, 2016). A large-scale global epidemiological survey showed that the prevalence rate of MCI in the elderly ≥ 60 years old was 15–20% (Petersen, 2016), and MCI is considered to be a high-risk disease transformed into Alzheimer’s disease (AD) and a precursor stage of AD (Petersen et al., 2018). Kluger et al. found that 2/3 of AD patients developed from MCI (Egerházi et al., 2008). Cognitive impairment diseases such as AD seriously affect an individual’s life and bring heavy economic burden to society.

According to China Association for Alzheimer’s Disease, intervention during the MCI stage can effectively slow down or stop the progression of AD (Alzheimer’s Association, 2018). As for the therapeutic effect of MCI, controversy remains at home and abroad, and no clear conclusion has been reached thus far. Transcranial direct current stimulation (tDCS) is a non-invasive neuromodulating technique, which may play a role in regulating brain function by changing cortical excitability, increasing synaptic plasticity, affecting cortical excitation/inhibition balance, changing local cerebral blood flow, and regulating the connection between local cortex and brain network (Mingyu et al., 2017; Yadollahpour and Yuan, 2018). Recent evidence has demonstrated the therapeutic efficacy of tDCS for different disorders including depression, addiction, cognitive impairments, stroke, Parkinson’s disease, cognitive impairment caused by cerebral infarction, and other disorders (Gandiga et al., 2006; Cosmo et al., 2015; Kunze et al., 2016; Yadollahpour et al., 2017b; Yadollahpour and Yuan, 2018; Yuan et al., 2018). Moreover, tDCS has been reportedly capable of improving different cognitive functions in patients as well as healthy individuals (Galea et al., 2009; Agarwal et al., 2013; Wolkenstein and Plewnia, 2013; Feeser et al., 2014; Ferrucci and Priori, 2014; Yadollahpour et al., 2017a; Nejati et al., 2018). In the current literature on the effectiveness and efficacy of tDCS on MCI patients, the findings are promising though controversial (Murugaraja et al., 2017; Inagawa et al., 2019; Holczer et al., 2020; Liu et al., 2020; Manenti et al., 2020; Chu et al., 2021; Ciullo et al., 2021). The findings of the studies have demonstrated that tDCS can improve the memory and cognitive function of MCI patients to varying degrees (Murugaraja et al., 2017; Cruz Gonzalez et al., 2018; Holczer et al., 2020; Manenti et al., 2020). Episodic memory is the earliest memory system to decline in MCI and AD patients, and the temporal lobe is closely related to episodic memory and plays an important role in memory consolidation (Jamil et al., 2017). Therefore, this study investigated whether tDCS over the temporal lobe could improve episodic memory of MCI patients, and explored its mechanism from the perspective of neuroelectrophysiology.

## Materials and Methods

All experimental procedures and study design were approved by local ethics committee of Wuxi Tongren Rehabilitation Hospital, Wuxi, Jiangsu, China (Ethics Code: WXMHCIRB2021LLky086), which were in complete accordance with the regulations and guidelines of human studies set by the Helsinki Declaration of 1975, as revised in 2014 (General Assembly of the World Medical Association, 2014). Following the enrollment, the main objectives of the study along with possible benefits and risks were clearly explained to all participants before start of the experiments. The informed consent was obtained from all patients for participation in this study.

### Inclusion and Exclusion Criteria

The inclusion criteria of the MCI group referred to Peterson and the Diagnostic and Statistical Manual of Mental Disorders (DSM-V) based on specific situations: (Petersen, 2016) age ≥ 50 years; (Petersen et al., 2018) the principal complaint or reliable insider confirmed that the cognitive impairment existed for more than 3 months; (Egerházi et al., 2008) Clinical Dementia Rating Scale (CDR) = 0.5; (Alzheimer’s Association, 2018) Montreal Cognitive Assessment Scale (MoCA) < 26 (MoCA score plus 1 point for those with schooling years ≤ 12 years); (Yadollahpour and Yuan, 2018) cognitive impairment had not yet reached the standard of dementia; (Mingyu et al., 2017) right handedness; (Yadollahpour et al., 2017b) no hearing impairment; and (Cosmo et al., 2015) be informed of this study and signed consent. Exclusion criteria: (Petersen, 2016) cognitive impairment caused by other reasons, including consciousness disorders, epilepsy, and psychiatric diseases caused by vascular causes; (Petersen et al., 2018) serious medical diseases complicated with important organs; (Egerházi et al., 2008) a history of head trauma; (Alzheimer’s Association, 2018) taking special drugs; and (Yadollahpour and Yuan, 2018) not cooperating with the treatment process.

### General Data

Participants of this study were selected from the patients referred to Wuxi Mental Health Center, Wuxi, Jiangsu, China from January 2019 to December 2020. A total of 40 patients who met the MCI inclusion criteria were selected for this study. Based on the random number table and envelope concealment grouping method, they were divided into the tDCS group and the sham group, with 20 cases in each group. Both the subjects and evaluators were unaware of the groups. Table 1 shows that there was no significant difference between the two groups in gender, age, schooling years, and MoCA score (all *P* > 0.05).

**TABLE 1:** Comparison of demographic information and basic clinical data between the two groups.

Group	Gender		Age (years)	Schooling years (years)	MoCA
	M	F			
Sham group	9	11	65.15 ± 6.16	9.90 ± 3.19	21.40 ± 2.64
tDCS group	13	7	63.20 ± 6.98	11.15 ± 2.96	22.20 ± 2.48
*T* or *χ^2^*	1.616		0.937	−1.284	−0.986
*P*	0.204		0.355	0.207	0.330

*M, Male; F, female. Data are presented as mean ± standard deviation (x ± s).*

### Methods

#### Therapy

We used IS300 type intelligent electrical stimulator (Sichuan Zhineng Electronics Industrial Co., Ltd., Chengdu, China) with electrode size of 7 cm × 5 cm. The stimulation site needed to be wetted with physiological saline before treatment. Body surface stimulation sites: The body surface positioning was in accordance with the international 10–20 electroencephalogram (EEG) system. The anode was placed in the left temporal area (T3), and the cathode electrode was placed contralaterally in the right shoulder deltoid muscle. Patients in the tDCS group received tDCS real stimulation with current intensity of 2.0 mA for 20 min once a day for 5 consecutive days. Patients in the sham group received tDCS pseudo stimulation: first, they were given a short current stimulation of 2.0 mA for 30 s, making the patients have the same subjective feeling as the real stimulation; then, the electric stimulation intensity was adjusted to be 0 mA, with each pseudo stimulation lasting for 20 min, once a day for 5 days (Lee et al., 2018).

#### Evaluation Indexes

To assess the primary and secondary outcomes of this study, we used three types of assessment tools, explained below.

(1) MoCA: The MOCA scale evaluates impairment from aspects such as memory, visual space, executive function, speech fluency, and abstract thinking. It can well reflect the characteristics of impairment in multiple fields and exhibits strong sensitivity to MCI.

(2) Wechsler Memory Scale (WMS): WMS is a commonly used memory scale worldwide with high sensitivity to memory impairment. The WMS we used was the Wechsler Memory Scale-Revised of China (WMS-RC), which includes experience, orientation, mental control, picture memory, recognition, visual regeneration, associative learning, tactile memory, logical memory, and memory span, a total of 10 points test. A rough score was obtained for each test. The rough score of each test was converted into scale scores, and the scale scores except experience and orientation were added up. Memory quotient (MQ) was obtained by consulting the corresponding table, and MQ represents the overall memory function. WMS-RC lacks the evaluation of delayed memory, so it cannot be used to accurately evaluate episodic memory. Therefore, according to Russell’s method (Russell, 1997), visual regeneration and logical memory were taken to represent non-verbal and verbal memory, respectively, and delayed memory was completed after 30 min, which was expressed with visual regeneration-delay and logical memory-delay. Similarly, the obtained rough scores were converted into scale scores, but they were not added to the total MQ score. The scores MQ ≥ 80 were considered as normal.

(3) Event-related potential (ERP) P300: Event related potential is a special brain evoked potential, in which auditory evoked P300 is proved to be closely related to complex cognitive activities. Amplitude and latency are its main evaluation indexes used to diagnose, predict, and observe the curative effect of MCI patients at present, considered to have high sensitivity and specificity (Picton et al., 2000). Therefore, we used them to provide a quantitative objective basis for the evaluation of the curative effect of MCI patients. Test methods: Stellate-64 lead paperless digital EEG/evoked potential instrument produced by Stellate Company of Canada was used to complete the test. The disc electrodes used were from ECI Company of Denmark. The electrodes were placed at Fz, Cz, Pz, C3, and C4 points according to the international 10–20 system of the International Electroencephalogram Society. The earth electrode was placed on the forehead, and the reference electrode was placed on the right mastoid process. The test was conducted in a quiet and comfortable environment with appropriate temperature. Blinking was avoided as much as possible. The subjects received short tone burst stimulation in both ears and made button response to target stimulation. EEG waves were recorded using HARMONIE software. BESA5.1 was used for offline analysis. The analysis time was 1,000 ms, and the artifact waveforms caused by eye movement or other reasons were deleted.

The above evaluation indexes were evaluated before treatment, after 5 days of treatment, and 4 weeks after completion of treatment. The evaluation was performed by the same psychometric physician who was unaware of the subjects’ treatment regimen.

### Statistical Analysis

Statistical Package for the Social Sciences (SPSS version 22.0, Windows) was used to analyze the data. The data were tested by normality and expressed in *x* ± s. *T*-test for two independent samples was used for the data conforming to normal distribution, and rank sum test for two samples was used for the data not conforming to normal distribution. General linear correlation analysis was applied. For all statistical analyses, the *P* < 0.05 values were considered as statistically significant.

## Results

During the treatment, there were two cases of skin itching and one case of prickling sensation in the tDCS group, and there was one case of skin itching in the sham group. After explanation and emotional comfort by treatment staffs, all patients completed the experiment. In the tDCS group, 1 patient had skin redness at the treatment site, which was recovered within 2 h after treatment, and the experiment was completed. None of the patients had headache, nausea, insomnia, anxiety, epilepsy, or other adverse reactions.

### Comparison of Different Cognitive Scale Scores Between Two Groups Before Treatment, After 5 Days of Treatment, and 4 Weeks After Completion of Treatment

Table 2 shows the comparisons of different cognitive functions and WMS-RC scores at each time point between the two groups. Data are presented as mean ± standard deviation. Table 2 shows that in the sham stimulation group, the scores of each scale had no significant changes after 5 days of treatment or 4 weeks after the end of treatment compared to before treatment (all *P* > 0.05).

**TABLE 2:** Comparison of cognitive scale scores at each time point between the two groups.

Groups	Time	MoCA	MQ	Mental control	Picture memory	Recognition	Visual regeneration	Associative learning	Tactile memory	Logical memory	Memory span	Visual regeneration delay	Logical memory-delay
Sham group (*n* = 20)	Before treatment	21.40 ± 2.64	62.05 ± 6.07	24.30 ± 3.59	4.85 ± 1.31	6.90 + 1.45	4.45 ± 1.40	5.50 ± 2.33	5.65 ± 2.11	6.05 ± 1.93	6.90 ± 1.77	4.45 ± 1.36	5.45 ± 1.64
	After 5 days of treatment	21.10 ± 2.34	64.25 ± 5.58	24.50 ± 2.80	4.65 ± 0.99	7.70 ± 1.53	4.40 ± 1.27	6.15 ± 2.64	6.45 ± 2.82	5.65 ± 2.01	6.45 ± 1.50	4.40 ± 1.60	5.05 ± 1.82
	4 weeks after completion of treatment	21.50 ± 2.63	62.75 ± 5.71	22.95 ± 3.05	4.70 ± 1.17	7.45 ± 1.64	4.05 ± 1.19	6.15 ± 2.62	5.80 ± 2.26	5.30 ± 1.87	6.05 ± 1.88	3.90 ± 1.41	4.85 ± 1.73
tDCS group (*n* = 20)	Before treatment	22.20 ± 2.48	64.00 ± 6.46	24.45 ± 3.44	4.25 ± 1.07	7.20 ± 1.77	4.40 ± 1.47	5.80 ± 2.38	5.55 ± 2.42	6.25 ± 1.94	6.90 ± 1.45	4.40 ± 1.43	5.80 ± 1.82
	After 5 days of treatment	22.15 ± 2.48	69.20 ± 6.71*^Δ^	24.55 ± 2.72	5.60 ± 1.19**^ΔΔ^	7.75 ± 1.89	6.25 ± 2.07**^ΔΔ^	6.80 ± 2.76	6.15 ± 2.23	8.65 ± 2.16**^ΔΔ^	9.15 ± 1.79**^ΔΔ^	6.35 ± 1.95**^ΔΔ^	8.05 ± 2.28**^ΔΔ^
	4 weeks after completion of treatment	22.25 ± 2.95	68.85 ± 6.43*^ΔΔ^	23.20 ± 3.00	6.45 ± 1.64**^ΔΔ^	7.60 ± 1.82	6.15 ± 2.18**^ΔΔ^	6.50 ± 2.50	6.15 ± 2.21	8.50 ± 2.14**^ΔΔ^	8.75 ± 1.94**^ΔΔ^	5.80 ± 2.19*^ΔΔ^	7.70 ± 2.06**^ΔΔ^

Compared with before treatment: *P < 0.05, **P < 0.01; Compared with sham group: ^Δ^ P < 0.05, ^ΔΔ^ P < 0.01. Data are presented as mean ± standard deviation (x ± s).

In contrast, in the tDCS group, MQ score after 5 days of treatment was improved compared with before treatment, and the difference was statistically significant (*P* < 0.05), and scores of picture memory, visual regeneration, logical memory, memory span, visual regeneration-delay, and logical memory-delay were also considerably improved (all *P* < 0.01). Similarly, 4 weeks after the end of treatment, MQ and visual regeneration-delay scores in the tDCS group were improved compared to before treatment, with the difference statistically significant (both *P* < 0.05), and scores of picture memory, visual regeneration, logical memory, memory span, and logical memory-delay were also considerably improved (all *P* < 0.01). In addition, there were no significant differences in scores of all scales between the two time points after treatment.

As for the inter-group comparison, there was no statistically significant difference in each score between the two groups before treatment (all *P* > 0.05). After 5 days of treatment, MQ score of the tDCS group was higher than that of the sham group, and the difference was statistically significant (*P* < 0.05). Four weeks after the end of treatment, MQ score was remarkably higher than that of the sham group, and the difference was more significant (*P* < 0.01). Either after 5 days of treatment or 4 weeks after the end of treatment, the scores of picture memory, visual regeneration, logical memory, memory span, visual regeneration-delay, and logical memory-delay in the tDCS group were significantly higher than those in the sham group (all *P* < 0.01).

### Comparison of P300 Amplitude and Latency Between the Two Groups Before Treatment, After 5 Days of Treatment, and 4 Weeks After the Completion of Treatment

Table 3 shows that in the sham group, P300 amplitude and latency were not significantly different from those before treatment, after 5 days of treatment, and 4 weeks after the end of treatment (both *P* > 0.05). In the tDCS group, the amplitude was significantly increased after 5 days of treatment and 4 weeks after the end of treatment, and the P300 latency was significantly shortened (all *P* < 0.01). However, the amplitude and latency had no statistically significant differences between the two time points after treatment (both *P* > 0.05). As for the inter-group comparation, no significant differences were observed in amplitude and latency between the two groups before treatment (both *P* > 0.05). After 5 days of treatment and 4 weeks after the completion of treatment, the amplitude of the tDCS group was significantly higher and the latency was significantly shorter than that of the sham group (all *P* < 0.01).

**TABLE 3:** Comparison of P300 indexes at each time point between the two groups.

Groups	Time	P300 amplitude (ms)	P300 latency (uV)
Sham group (*n* = 20)	Before treatment	4.01 ± 1.51	336.00 ± 31.31
	After 5 days of treatment	4.58 ± 1.70	334.00 ± 34.32
	4 weeks after completion of treatment	4.49 ± 1.64	329.25 ± 42.62
tDCS group (*n* = 20)	Before treatment	3.66 ± 1.44	332.25 ± 26.13
	After 5 days of treatment	7.36 ± 1.95**^ΔΔ^	277.25 ± 28.54**^ΔΔ^
	4 weeks after completion of treatment	7.26 ± 1.83**^ΔΔ^	276.40 ± 26.75**^ΔΔ^

Data are presented as mean ± standard deviation (x ± s). Compared with before treatment: 1, **P < 0.01; Compared with sham group: ^ΔΔ^P < 0.01.

### Correlation Between Memory Quotient (MQ) Difference and P300 Amplitude or Latency Difference Before Treatment and After 5 Days of Treatment in the Transcranial Direct Current Stimulation Group

According to Table 4, before treatment and 5 days after treatment, P300 amplitude difference was positively correlated with MQ difference (both *P* < 0.05), and P300 latency difference was also positively correlated with MQ difference with statistical significance (both *P* < 0.01), suggesting that in the tDCS group, the greater the increase of amplitude or the shortening of latency after treatment, the greater the increase of MQ score.

**TABLE 4:** Correlation between MQ difference and P300 amplitude or latency difference before and after treatment (*r*).

Item	P300 amplitude difference	P300 latency difference
MQ difference	0.563*	0.698**

*P < 0.05, **P < 0.01.

## Discussion

Episodic memory mainly refers to remembering specific events at a certain time and a certain place in the past; it depends on a series of psychological processes, including coding, and storage and retrieval of internal or external information (Squire, 2004). The recall of five words in the MoCA scale, picture memory, visual regeneration, and logical memory in WMS are all episodic memory. Episodic memory can be manifested as the ability to learn new knowledge, which is anterograde memory, or the ability to extract new information, which is retrograde memory. From Table 2, we found that the scores of picture memory, visual regeneration, logical memory, visual regeneration-delay, and logical memory-delay in the tDCS group after 5 days of treatment were higher than those before treatment. Picture memory, visual regeneration, and logical memory are immediate memory, which can reflect anterograde memory, and visual regeneration-delay and logical memory-delay are delayed memory, which can reflect retrograde memory. This experiment comprehensively reflected the improvement of episodic memory at the verbal and non-verbal levels.

The temporal lobe, a key structure for learning, recognition, and recall, is impaired in MCI and AD. The medial temporal lobe (MTL), especially the hippocampus, plays a central role in episodic memory function (Karantzoulis and Galvin, 2011). Changes of recall delay in MCI and AD patients reflect impaired functions of entorhinal cortex and hippocampal cortex (Liu et al., 2020). Many studies abroad have shown that tDCS can improve the memory function of MCI and AD patients. Chi et al. (2010) found that the visual long-term memory of subjects was significantly improved after tDCS in the anterior temporal lobe. Yun et al. (2016) improved subjective memory satisfaction and immediate memory of MCI patients by tDCS stimulation. Consistent with previous studies, in this study, tDCS stimulation in the left temporal region improved the episodic memory in MCI patients. In Table 2, MQ in the tDCS group increased after treatment, suggesting that the overall memory function was improved after tDCS treatment, but MoCA score did not increase significantly, the possible reason of which may be that the test reflecting immediate and delayed memory in MoCA was relatively simple. The test may have a certain learning effect and cannot accurately reflect the changes of memory after tDCS treatment. The scores of memory span increased after treatment, suggesting that tDCS also improved the attention and executive function of MCI patients (Table 2). In addition to the impairment of memory function, executive function was also the earliest and most common damage field in MCI (Guo et al., 2010).

Event-related potential P300 is the bioelectrical activity generated by the nerve center during the process of sensing information stimulation, P300 latency represents the speed of nerve transmission in response to stimulation, and amplitude is a measure of how much the brain region is activated during information processing; longer P300 latency and lower P300 amplitude indicate worse recognition of external stimuli and more serious cognitive dysfunction (Hedges et al., 2016; Tang et al., 2016). The results suggest that in the tDCS group, the P300 latency was shortened and the P300 amplitude was increased after 5 days of treatment, which reflected that the nerve conduction velocity became faster, and the activation of the brain area was enhanced when the brain responds to stimulation (Table 4). This effect can be attributed to the change of cortical excitability and increase of synaptic remodeling by tDCS. The same as AD, the pathological basis of MCI is the dysfunction of cholinergic, GABAergic, and glutamatergic systems, which leads to the damage of neural plasticity and the imbalance of brain activation, resulting in cognitive defects (Wang et al., 2009). Evidence shows that the activation of temporal cortical regions is reduced in patients with MCI and AD (Saunders and Summers, 2010), significant density reduction of soluble amyloid β level related synapsis and loss of presynaptic and postsynaptic components result in neuronal death and block long-term enhancement, and thus affect memory function (Peelle et al., 2014). tDCS current can affect the resting membrane potential of neurons (Mingyu et al., 2017). After stimulating the temporal region with anodic current, we promoted neuronal potential depolarization, enhanced neuronal excitability, regulated the expression of N-methyl-D-aspartate receptor and the release of γ-aminobutyric acid, produced long-term enhancement, and caused synaptic remodeling (Stagg and Nitsche, 2011). By enhancing the synaptic efficiency, the signal transduction efficiency of the neural pathway was improved, thus the memory function was enhanced. Table 4 shows the correlation between MQ difference and P300 amplitude or latency difference after 5 days of treatment. The results showed that the greater the amplitude increase and latency reduction, the more obvious the improvement of memory function.

This study further extended the observation time to 4 weeks after the end of treatment. The results in Tables 2, 3 showed that the scores of MQ, picture memory, visual regeneration, logical memory, visual regeneration-delay, logical memory-delay, and memory span were still improved compared with those before treatment, the P300 latency was shorter, and the amplitude was higher than that before treatment, but have no significant change compared to 5 days of treatment, which confirmed that the improvement effect of tDCS on memory and brain function did not weaken significantly until 4 weeks after treatment. It is proved that tDCS was not a short-term effect, and its post-stimulation effect lasted for at least 4 weeks, which is similar with the findings of Boggio et al. (2012).

## Conclusion

In conclusion, the results of this study suggested that tDCS can improve episodic memory in MCI patients. tDCS, as a neural regulation technology with high safety, good curative effect, low investment cost, and strong operability, has broad application prospects in the clinic. However, this study still has some limitations. First, the sample size was not large enough. A larger-scale experiment is needed to verify our conclusion. Second, the stimulation time of tDCS in this study was 5 days, and the efficacy tracking time was 4 weeks after the end of treatment. For future possible study, the follow-up observation period can be appropriately extended, and the stimulation time and cycle that can obtain the optimum enhancement effect can be determined through continuous repetition and comparison. In the future, we can combine functional magnetic resonance imaging, positron emission tomography-computed tomography and other imaging technologies to further clarify the treatment mechanism of tDCS and find a better stimulation mode.

## Data Availability Statement

The original contributions presented in the study are included in the article/supplementary material, further inquiries can be directed to the corresponding author/s.

## Ethics Statement

All experimental procedures and study design were reviewed approved by Local Ethics Committee of Wuxi Tongren Rehabilitation Hospital, Wuxi, Jiangsu, China (Ethics Code: WXMHCIRB2021LLky086). The patients/participants provided their written informed consent to participate in this study.

## Author Contributions

JG and ZL conceptualized the initial study design and critically reviewed the manuscript. JG, DL, ZL, YG, FQ, YW, and LT extracted the clinical data. All authors interpreted the data, provided constructive feedback during manuscript development, and read and approved the final manuscript.

## Conflict of Interest

The authors declare that the research was conducted in the absence of any commercial or financial relationships that could be construed as a potential conflict of interest.

## Publisher’s Note

All claims expressed in this article are solely those of the authors and do not necessarily represent those of their affiliated organizations, or those of the publisher, the editors and the reviewers. Any product that may be evaluated in this article, or claim that may be made by its manufacturer, is not guaranteed or endorsed by the publisher.
